# New approaches to recovery after stroke

**DOI:** 10.1007/s10072-023-07012-3

**Published:** 2023-09-11

**Authors:** Daniel S. Marín-Medina, Paula A. Arenas-Vargas, Juan C. Arias-Botero, Manuela Gómez-Vásquez, Manuel F. Jaramillo-López, Jorge M. Gaspar-Toro

**Affiliations:** Grupo de Investigación NeuroUnal, Neurology Unit, Universidad Nacional de Colombia, Bogotá, Colombia

**Keywords:** Neuronal plasticity, Biomedical technology, Rehabilitation, Stroke, Stroke rehabilitation

## Abstract

After a stroke, several mechanisms of neural plasticity can be activated, which may lead to significant recovery. Rehabilitation therapies aim to restore surviving tissue over time and reorganize neural connections. With more patients surviving stroke with varying degrees of neurological impairment, new technologies have emerged as a promising option for better functional outcomes. This review explores restorative therapies based on brain-computer interfaces, robot-assisted and virtual reality, brain stimulation, and cell therapies. Brain-computer interfaces allow for the translation of brain signals into motor patterns. Robot-assisted and virtual reality therapies provide interactive interfaces that simulate real-life situations and physical support to compensate for lost motor function. Brain stimulation can modify the electrical activity of neurons in the affected cortex. Cell therapy may promote regeneration in damaged brain tissue. Taken together, these new approaches could substantially benefit specific deficits such as arm-motor control and cognitive impairment after stroke, and even the chronic phase of recovery, where traditional rehabilitation methods may be limited, and the window for repair is narrow.

## Introduction

In clinical practice, the number of stroke patients is increasing due to the aging of the population and the high prevalence of cardiovascular comorbidities [1]. This is directly correlated with the growing number of stroke survivors who receive acute therapies, such as thrombectomy or pharmacological reperfusion. Early recognition of stroke has led to a larger population of patients who can survive and be discharged from hospitals with less severe neurological impairment. However, a significant proportion of patients do not receive acute therapies due to contraindications or being outside the reperfusion time window, and a significant proportion of patients survive with severe neurological deficits thanks to life-support systems. For these reasons, stroke is a major cause of disability worldwide and the leading neurological cause of lost disability-adjusted life years [2].

Traditionally, rehabilitation for patients with persisting deficits has focused on physical, occupational, and speech-language therapy, as well as the prevention of medical complications [3]. However, new rehabilitation strategies are now part of standard care and include activity-based therapy such as constraint-induced movement therapy, high-dose activity-based rehabilitation, high doses of task-specific training, mirror therapy, and environmental enrichment [4]. Restorative therapies targeting neuroplasticity mechanisms with recent technological advances have emerged as a promising tool for the integral care of patients with special needs. This review addresses the neural basis for stroke recovery and new approaches, including brain-computer interfaces (BCIs), virtual reality, robot-assisted rehabilitation, cell therapies, and brain stimulation. A comprehensive search was conducted in the PUBMED/Medline and SCOPUS databases for articles published in the last 10 years using the terms “robot/virtual reality/brain-computer interface/cell therapy/brain stimulation” combined with the terms “stroke/brain ischemia/rehabilitation/neuroplasticity” in English. Relevant articles and their bibliographic references were included based on the authors’ criteria for clinical practice relevance.

## Neuroplasticity after stroke

Neuroplasticity is the ability of the nervous system to modify and regenerate in response to new information or damage. Until recently, it was believed that neuroplasticity was nonexistent in adulthood. However, it has been found that neuroplasticity occurs spontaneously throughout life, although these changes are not sufficient to produce evident recovery after brain damage. Therefore, there are now several strategies available to enhance neuroplasticity, including both pharmacological and non-pharmacological interventions that are essential for post-stroke rehabilitation [5–7].

**Fig. 1:**
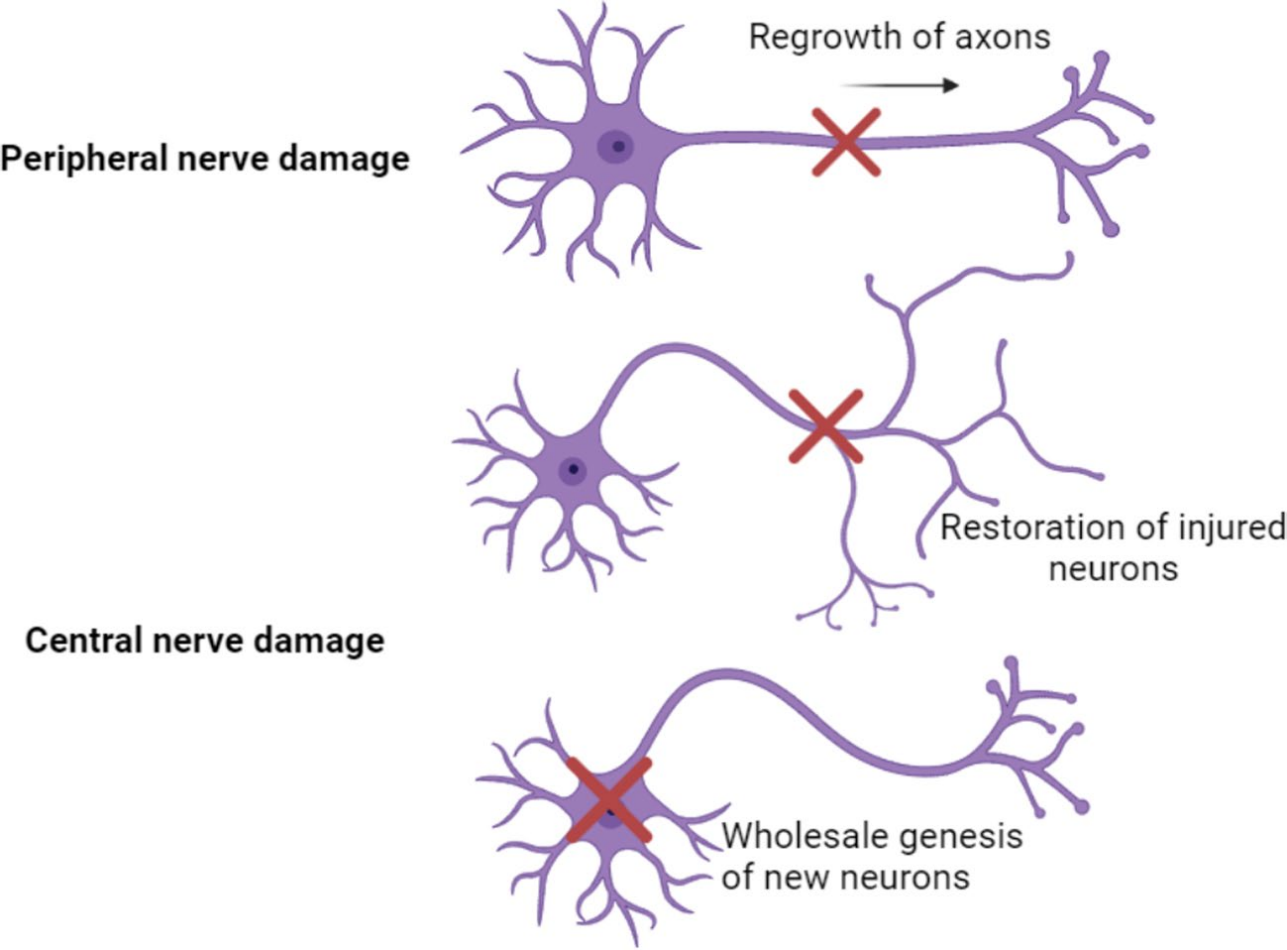
Mechanisms of neuroplasticity in healthy individuals

The current understanding of neuroplasticity is based on Hebb’s theory from 1949, which posits that repetitive stimulation of the postsynaptic neuron by the presynaptic neuron is necessary to increase synaptic efficacy [8]. There are three main mechanisms of neuroplasticity in healthy brains. The first is the regrowth of axons after peripheral nerve damage. The second is the restoration of injured central nerve cells through the growth of new dendrites, axons, and synapses from existing cell bodies. The third mechanism is the wholesale generation of new neurons, which occurs in two neurogenic regions: the subventricular zone and the dentate gyrus. In the subventricular zone, neuroblasts are generated and migrate along the rostral migratory path to the olfactory bulb, where they become granular and periglomerular interneurons involved in plastic processes of olfactory learning. In the dentate gyrus, stem cells give rise to neuronal precursors that mature and generate new granular neurons [9, 10] (Fig. 1).

**Fig. 2:**
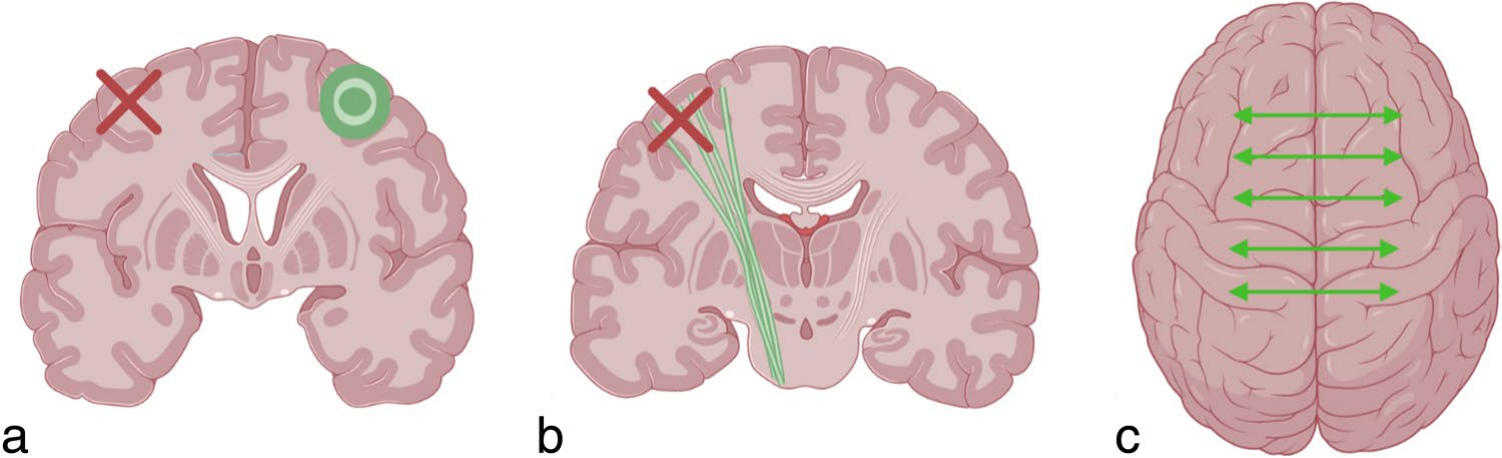
Neuroplasticity after stroke

Currently, three major mechanisms have been described for how neuroplasticity works after a stroke. These mechanisms begin in the early stages after the event and continue for at least 3–6 months, leading to the reorganization of neural connections [11, 12]. The first mechanism involves increased functional activity in the somatosensory system on the opposite side of the brain from the infarction, as well as recruitment from distant cortical regions connected to the affected area [13, 14] (Fig. 2a). The second mechanism involves the improvement of the structural integrity of the corticospinal tract on the same side of the brain as the infarction [15] (Fig. 2b). The third mechanism involves the restoration of interhemispheric functional connectivity and the network of the sensorimotor cortex on both sides of the brain [16] (Fig. 2c). As a result, there is a reallocation of functions whose primary representation has been damaged [11].

## Brain-computer interfaces

A BCI is a modern tool that allows for the transduction of brain signals into computational commands, enabling the control of technological devices. The positive effects of BCI have been discovered to mainly stimulate neuroplasticity and assist patients with loss of motor function, leading to hopeful clinical implications for post-stroke patients and individuals with other neurological diseases [17–20]. The operation of BCI is based on three main components: signal reception, signal processing, and generation of a specific response in a machine. Multiple methods for developing each of these components have been reported [18, 20–23].

There are two methods for the reception of brain signals, invasive and non-invasive. The invasive method, which may produce a cleaner signal, carries the risks of surgery and is not always the most suitable for clinical use. There are three types of invasive electrodes, including cortical surface microelectrodes (ECoG), cortical penetrating microelectrodes, and deeply penetrating electrodes, which record different characteristics of brain action potentials. Non-invasive approaches, such as electroencephalography (EEG), are more commonly used and have the most scientific evidence to support their effectiveness. However, alternative methods like near-infrared spectroscopy have also been reported [18, 20].

The second step in the BCI process involves filtering the received brain signals using specific techniques to enable interpretation. The commonly used techniques include sensorimotor rhythms, slow cortical potentials, event-related potentials/P300, and visual evoked potentials. The filtered signals are then transformed into voltage/time frequencies using processing techniques such as Fourier, common spatial filter, and wavelet transform. These signals are further analyzed through classification algorithms, which generate a specific command for the machine to execute [18, 20].

The final step in BCI involves generating a response using a device that is programmed to receive the signal and perform a function, such as basic motor patterns to improve rehabilitation and quality of life [18, 20]. In stroke patients, BCI-assisted rehabilitation is an alternative approach that aims to stimulate neuroplasticity by manipulating or self-regulating brain activity, resulting in motor cortical reorganization and changes in motor activation ipsilateral and contralateral to the lesion. BCI has been found to be superior to several types of conventional therapy, as reflected in motor connectivity in fMRI, increased event-related desynchronization activity in the EEG, and increased volitional contraction in electromyographic activity in affected muscles. These, along with other changes not measurable with clinical scales, suggest a promising mechanism for managing these patients [17]. However, the physiological basis of this recovery has not yet been fully elucidated, nor have the factors that influence better outcomes with these therapies, making it an area with great potential for future applications in clinical settings [18, 24, 25].

## Robot-assisted rehabilitation and virtual reality

Virtual reality (VR) therapy and robot-assisted therapies (RAT) are emerging technologies that have shown promise as alternative treatments for improving motor function and quality of life [26–29]. VR therapy involves the use of an interface between the computer and the patient, using both hardware and software to simulate interactions with the environment. This allows for the creation of sensory connections that closely mimic reality, with the added benefits of simultaneous task execution and immediate feedback [26, 30]. VR systems are specifically designed to aid in the rehabilitation of patients with neurocognitive impairments, using specialized interfaces that focus on developing functional skills that can be applied in the real world [30, 31].

RAT have shown promise in training lost motor function or compensating for lost skills after stroke. The use of robots in stroke rehabilitation has led to positive trends in motor improvement. For instance, an exoskeleton or a robotic hand can be used to rehabilitate gait and upper limb motor function, respectively. These devices support the patient’s movements in different axes and provide better control and monitoring for specific tasks and patient needs. However, the impact of RAT depends on factors such as the type of support provided to the limb, the patient’s ability to execute the movement, the duration of robot support, and the type of exercise. Additionally, limited research has been conducted on the impact of therapy performed solely with robots, and it is more feasible to use RAT in combination with other techniques such as VR therapy to improve overall motor and cognitive performance [32, 33].

In VR therapy, there are different levels of immersion that can be utilized based on the patient’s needs. Skillful immersion focuses on improving specific motor skills, while strategic immersion aims to improve higher-level cognitive skills such as decision-making and problem-solving. Narrative immersion uses storytelling to provide a context for the exercises and increase engagement, while spatial or total immersion involves creating a completely immersive virtual environment for the patient to interact with. Total immersion is the most immersive type of VR therapy, providing the patient with a fully immersive experience where they are transported to a virtual world or scenario. This can be helpful in providing a distraction from the real world, while still allowing the patient to engage in meaningful activities that improve physical and cognitive function. The use of a virtual scenario with a specific story can also provide emotional and psychological benefits for the patient. Additionally, performance can be monitored and measured in real time, and feedback can be given to the patient to help them improve [26, 27, 30].

**Fig. 3:**
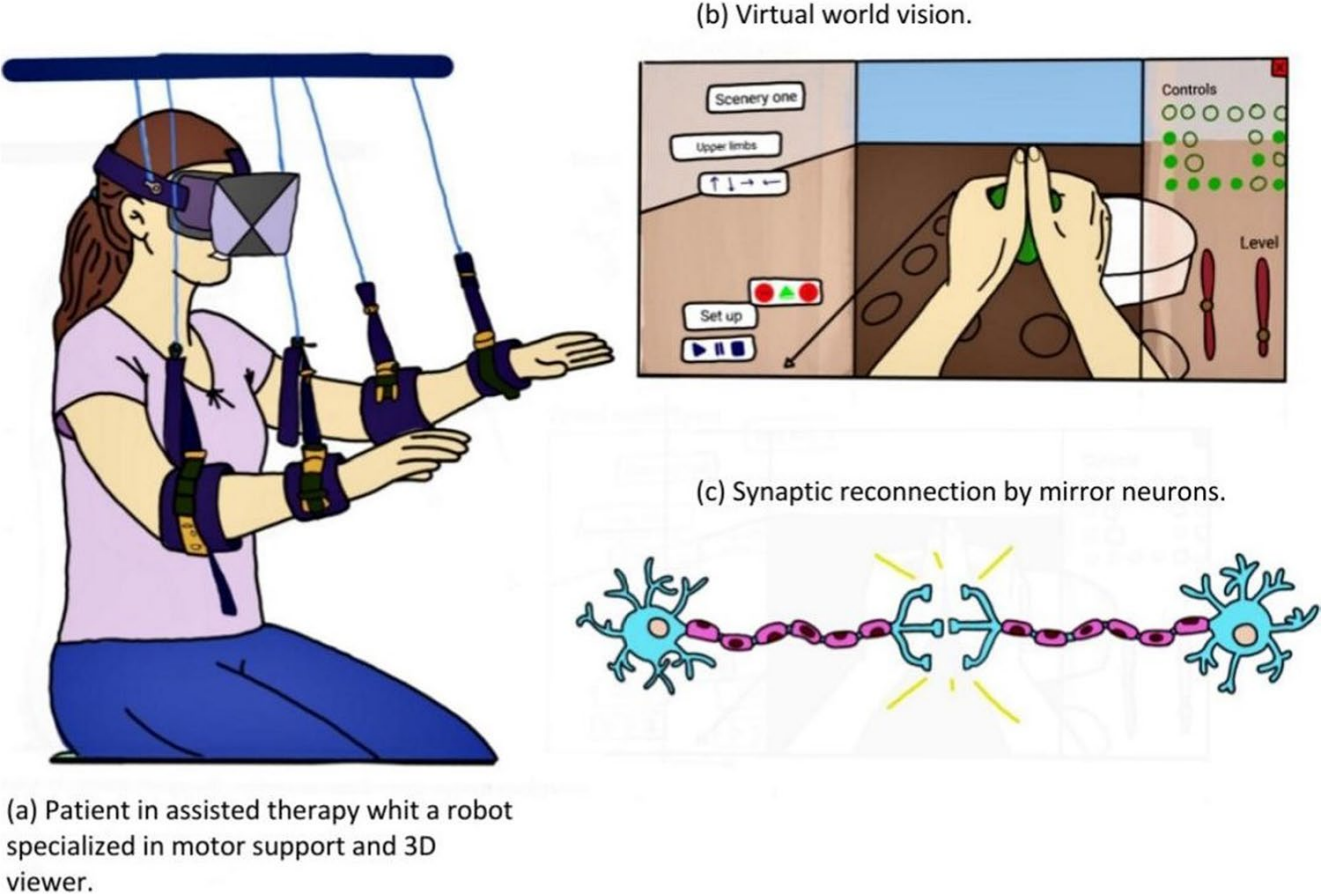
Setup and scenario of VR-Rehab system: **a** the patient can be seen wearing a head-mounted display (HMD) headset and a 3D viewer and monitored by two infrared cameras that track her exact position in the virtual world. The patient also has movement controllers in the form of purple bands on their arms, equipped with sensors that track their gestures and replicate them graphically within the virtual world. **b** The therapist can monitor the patient’s performance through the PC, which displays the virtual world. The illustration shows a typical exercise in which the patient must move a virtual ball to a virtual basket using their virtual upper limb. **c** Through immersion in virtual reality and performing these exercises, the patient’s mirror neurons are stimulated, which through repetition contributes to the formation of new synaptic connections and the overall improvement of their physical condition

VR therapy is an effective way to provide game-based training tasks to patients with motor deficits. These tasks, such as grasping and releasing a virtual ball, are designed to stimulate and activate mirror neurons. As shown in Fig. 3, patients wear a head-mounted display and use movement controllers to interact with the virtual environment. After 4 weeks of treatment, mirror neuron VR rehabilitation (MNVR-Rehabs) has been shown to promote neuroplasticity in injured brain areas and lead to functional performance improvement. Resting fMRI and Fugl-Meyer assessment support the effectiveness of MNVR-Rehabs in reorganizing brain and motor function [34].

The results of rehabilitation using VR and robot-assisted therapies can be influenced by differences in intensity, duration, frequency, and personalization. These modalities do not impact overall cognitive function, verbal fluency, or attention compared to the control groups, but they do lead to significant improvements in dynamic balance, executive function, memory, motor function, visuospatial ability, and quality of life [27, 35]. Balance improvement is closely tied to the visual, vestibular, and somatosensory systems, which can be targeted through VR therapy to stimulate and practice postural control in a safe and controlled environment, ultimately reducing fall risk and enhancing cognitive processes [36]. Multidisciplinary rehabilitation that includes immersion therapy using robot assistance can further improve gait control parameters and offer greater control during therapy [26, 28, 32].

Furthermore, the use of robot-assisted therapy has been shown to result in improvements in upper limb motor function, spasticity, and overall functioning, leading to better performance of daily activities. Additionally, patients who received RAT showed better results in cognitive evaluations compared to the control groups. While VR therapy has shown potential for future treatments in stroke rehabilitation, it did not show statistically significant differences in cognitive function compared to the control groups [31].

## Cell therapies

The brain’s limited repair capacity has led to the development of new strategies for enhancing brain plasticity, including cell therapy. Stem cells have the potential to differentiate into multiple mature and immature cell types, and therefore have the ability to promote regeneration in damaged brain tissue [37, 38]. Various cell lines have been explored for the treatment and rehabilitation of stroke, including embryonic stem cells (ESCs), neural stem cells (NSCs), bone marrow mononuclear cells (BMMCs), mesenchymal stem cells (MSCs), induced pluripotent stem cells (iPSCs), and others. Among these, MSCs are particularly promising due to their low immunogenicity, easy availability, and positive results in animal models [37, 38]. MSCs are characterized by their adherence to plastic matrix-cultures, fibroblast-like morphology, expression of surface antigens (CD44, CD90, CD29), and ability to differentiate into mesodermal and neural cells [37, 39].

Multiple animal studies have demonstrated that stem cell therapy can improve post-stroke functionality by stimulating various mechanisms, including neuron replacement, trophic stimulus, angiogenesis promotion, remyelination induction, and cell protection [38]. Three main theories have been proposed to explain MSC-mediated brain repair, including “trans”-differentiation, cell fusion, and the paracrine effect, through the release of soluble trophic factors [37, 40]. While there is evidence for all of these phenomena, it is uncertain how much each contributes to rehabilitation and functional improvement [40]. Among these, the release of trophic factors seems to play a significant role in the MSC-mediated brain repair response after stroke. The effects of these factors can be classified as angiogenic, neurogenic, protective, synaptogenic, and prevention of pathological scarring [40, 41]. The immune regulation and tolerance provided by these soluble factors can prevent and regulate the secondary inflammatory response, which can be more harmful than protective. In addition, in vitro studies have shown that MSCs can increase the expression of angiogenic factors such as vascular endothelial growth factor (VEGF) and brain-derived neurotrophic factor (BDNF). Exosomes, which contain DNAs, RNAs, mRNAs, peptides, and other bioactive molecules, have also been found to play a crucial role in MSC-mediated brain repair by stimulating nerve repair, reducing inflammation, and promoting successful remodeling [42]. In fact, the delivery of exosomes may be comparable to MSC transplantation.

There have been human studies on stem cell therapy [43]. One study published in 2019 focused on the safety of intravenous doses of allogeneic ischemia-tolerant mesenchymal stem cells and the potential behavioral changes after therapy. The study concluded that intravenous transfusion of allogeneic ischemia-tolerant mesenchymal stem cells was safe for patients with chronic stroke and significant functional deficits, and suggested possible behavioral improvements. However, the results should be evaluated further with a randomized, placebo-controlled study [44]. Another study, called the PISCES-2 study, used direct intracerebral implantation of neural stem cells by stereotaxic injection to the putamen ipsilateral to the cerebral infarct. The study aimed to observe upper limb movement improvement, but the clinical enhancement was only seen in those with residual upper limb movement at baseline [45]. The RECOVER stroke trial used internal carotid infusion of autologous bone marrow-derived aldehyde dehydrogenase-bright stem cell and found no allergic reactions or adverse events related to the cell therapy, but there was no significant difference between the intervention and placebo groups in the modified Rankin scale (mRS) at 3 months [45, 46]. Another randomized clinical trial, called the ISIS-HERMES study, evaluated the safety, efficacy, and feasibility of intravenous autologous bone marrow-derived MSCs infusion in subacute stroke patients (< 2 weeks after diagnosis). The study found no differences in the Barthel Index, NIHSS, and mRS, but there were significant improvements in motor recovery evidenced with motor-NIHSS and motor-Fugl-Meyer score. The study also observed that intravenous MSCs treatment was safe [47]. These studies indicate that we are getting closer to identifying a consistent, secure, and efficient method for using cell therapy in stroke recovery and rehabilitation, particularly with MSCs. However, there is still a need for further research to demonstrate a strong effect in actual clinical practice.

## Brain stimulation

Non-invasive brain stimulation (NIBS) is a widely used technique in many fields, particularly in stroke rehabilitation [48]. Both transcranial magnetic stimulation (TMS) and transcranial direct current stimulation (TDCS) have demonstrated improved functional outcomes and responses in individuals who have experienced a stroke. These techniques have been observed to induce changes in long-term neuroplasticity, modulating local and distant networks that underlie various clinical symptoms resulting from stroke [49–52].

TMS is a technique that uses electromagnetic induction to create an electric current which can modulate the electrophysiological activity of cells, leading to neuron depolarization and action potential. The frequency of the pulses can alter cortical activity by reducing the permeability of the neurovascular complex, thereby improving cerebral perfusion and angiogenesis [53]. TMS can also modulate cortical excitability (CE), with low-frequency TMS (<1 Hz) decreasing excitability (commonly used in the contralateral hemisphere to inhibit activity) and high-frequency TMS (>1 Hz) increasing excitability (used in the affected area to stimulate activity) [54, 55]. The primary motor cortex, parietal cortex, and Broca-Wernicke areas are commonly treated with TMS [56–59]. There is a subtype of repetitive TMS called theta burst stimulation (TBS), which can enhance in intermittent mode (iTBS) or depress in continuous mode (cTBS) and has been used in stroke recovery; beyond its original description in primary motor areas, there are publications on TBS in the cerebellum, given its parietal-frontal connections, showing improvement in motor learning [60, 61].

TDCS generates a weak direct electrical constant current (1–2 mA) that modulates CE, stimulates neuroplasticity, improves local blood flow, and has an effect over nearby areas and connectivity; the anode depolarizes the resting potential (potentiating CE), while the cathode hyperpolarizes it (inhibiting CE) [62–65]. In studies using TDCS, current intensities of 1, 1.5, and 2 mA have been applied for a duration of 20 min. Anodic TDCS stimulates the affected primary motor cortex, while cathodic TDCS stimulates the contralateral primary motor cortex; high intensity and long duration TDCS have been found to yield better results [66].

TMS has been studied for its potential to aid in post-stroke rehabilitation in both acute and subacute cases. Various testing protocols for motor function, neglect, and aphasia have shown better recovery, although the results have been contradictory because the symptoms have not been compared between them or at the same time [67–69]. In contrast, TDCS has had minimal impact on post-stroke rehabilitation in the acute and subacute periods, possibly due to a lack of standardization in trials. However, in chronic scenarios, TDCS has been found to improve motor function as an adjuvant to other classical therapies [70, 71].

TMS is generally considered safe, but adverse events such as seizures, headache, cervicalgia, dizziness, and local discomfort have been reported in a low proportion of cases. Seizures are rare but more common with high-frequency TMS [72–74]. On the other hand, TDCS is better tolerated, with fewer effects such as skin redness, slight tingling, dizziness, and fatigue, depending on the current dose used [75]. In conclusion, NIBS holds promise as an adjuvant therapy for future research and possible translational applications in clinical scenarios [76], particularly for post-stroke rehabilitation [77].

## Future and perspectives

Traditionally, the phases of recovery after stroke have been divided into acute (first month), functional or subacute (6 months), and chronic or plateau (first year and thereafter) [78]. However, new approaches to stroke rehabilitation could be incorporated into these different phases and improve the outcomes of standard therapies. For example, cell therapy is a promising intervention for the acute and subacute phases, and may eventually become part of reperfusion therapies. Brain-computer interfaces, transcranial brain stimulation, robot-assisted therapy, and virtual reality can benefit not only the subacute phase but also the chronic phase of recovery, where classical rehabilitation may be limited and the time window for repair is narrow. However, these new approaches should be coupled with training and other restorative therapies for the best results.

The availability of rehabilitation services varies across countries, and many stroke patients suffer from delays and poor quality of rehabilitation services due to limited options and other public health barriers. Although the new technologies described in this paper hold great promise for stroke rehabilitation, they are still in the clinical phase of research and their availability is limited due to their high cost and complexity. However, given the high impact of stroke disability and associated costs, it is important to continue developing and implementing new strategies for stroke recovery.

## Conclusions

Stroke disability is an area of increasing interest, as recovery after stroke involves various neural mechanisms of plasticity that can be potentiated with both traditional and new rehabilitation therapies. The emerging approaches for stroke rehabilitation include brain-computer interfaces, robot-assisted therapy coupled with virtual reality, brain stimulation, and cell therapy. Although ongoing research is still exploring the impact of these modalities on stroke recovery, promising results suggest that these new therapies could lead to better functional outcomes for stroke patients.
